# Geraniol Potentiates the Effect of Fluconazole against Planktonic and Sessile Cells of Azole-Resistant *Candida tropicalis*: In Vitro and In Vivo Analyses

**DOI:** 10.3390/pharmaceutics16081053

**Published:** 2024-08-09

**Authors:** Gislaine Silva-Rodrigues, Isabela Madeira de Castro, Paulo Henrique Guilherme Borges, Helena Tiemi Suzukawa, Joyce Marinho de Souza, Guilherme Bartolomeu-Gonçalves, Marsileni Pelisson, Cássio Ilan Soares Medeiros, Marcelle de Lima Ferreira Bispo, Ricardo Sérgio Couto de Almeida, Kelly Ishida, Eliandro Reis Tavares, Lucy Megumi Yamauchi, Sueli Fumie Yamada-Ogatta

**Affiliations:** 1 Postgraduate Program in Microbiology, Department of Microbiology, State University of Londrina, Londrina 86057-970, Brazil; gislaine.srodrigues@uel.br (G.S.-R.); isabela.mcastro@uel.br (I.M.d.C.); paulo.guilhermeph@uel.br (P.H.G.B.); helena.tiemi.suzukawa@uel.br (H.T.S.); lionilmy@uel.br (L.M.Y.); 2 Postgraduate Program in Clinical and Laboratory Pathophysiology, Department of Pathology, Clinical and Toxicological Analysis, State University of Londrina, Londrina 86038-350, Brazil; 3 Department of Pharmaceutical Sciences, Federal University of Paraíba, João Pessoa 58059-900, Brazil; cassioism@hotmail.com; 4 Synthesis of Medicinal Molecules Laboratory, Department of Chemistry, State University of Londrina, Londrina 86057-970, Brazil; mlfbispo@uel.br; 5 Laboratory of Antifungal Chemotherapy, Institute of Biomedical Sciences, University of São Paulo, São Paulo 05508-900, Brazil; ishidakelly@usp.br; 6 Department of Medicine, Pontifical Catholic University of Paraná, Campus Londrina, Londrina 86067-000, Brazil; tavares.eliandro@gmail.com; 7 Laboratory of Molecular Biology of Microorganisms, Department of Microbiology, State University of Londrina, Londrina 86057-970, Brazil

**Keywords:** antibiofilm activity, Cdr1p, efflux pump activity, *Galleria mellonella* infection model, itraconazole resistance, in silico toxicity analyses, molecular docking, monoterpenoid, synergism

## Abstract

*Candida tropicalis* is regarded as an opportunistic pathogen, causing diseases ranging from superficial infections to life-threatening disseminated infections. The ability of this yeast to form biofilms and develop resistance to antifungals represents a significant therapeutic challenge. Herein, the effect of geraniol (GER), alone and combined with fluconazole (FLZ), was evaluated in the planktonic and sessile cells of azole-resistant *C. tropicalis*. GER showed a time-dependent fungicidal effect on the planktonic cells, impairing the cell membrane integrity. Additionally, GER inhibited the rhodamine 6G efflux, and the molecular docking analyzes supported the binding affinity of GER to the *C. tropicalis* Cdr1 protein. GER exhibited a synergism with FLZ against the planktonic and sessile cells, inhibiting the adhesion of the yeast cells and the viability of the 48-h biofilms formed on abiotic surfaces. *C. tropicalis* biofilms treated with GER, alone or combined with FLZ, displayed morphological and ultrastructural alterations, including a decrease in the stacking layers and the presence of wilted cells. Moreover, neither GER alone nor combined with FLZ caused toxicity, and both treatments prolonged the survival of the *Galleria mellonella* larvae infected with azole-resistant *C. tropicalis*. These findings indicate that the combination of GER and FLZ may be a promising strategy to control azole-resistant *C. tropicalis* infections.

## 1. Introduction

*Candida tropicalis* is a ubiquitous fungal species that can be found as a commensal on human skin, nails, and mucous membranes [1,2], as well as in the environment [3]. However, as an opportunistic microorganism, it is among the most prevalent species related to candidemia, with a high incidence in Asia and South America, including Brazil [4]. Importantly, *C. tropicalis* candidemia has been associated with high mortality rates, ranging from 26% to 40% in pediatric patients, and from 55% to 60% in adult patients [5,6]. Additionally, this species can cause infections in the upper respiratory, urinary, and gastrointestinal tracts [4,7].

*C. tropicalis* can express virulence factors that allow it to invade the host’s epithelial barriers and evade the host’s defenses, contributing to the pathogenesis of the infection [4,8]. Notably, this fungal species is capable of forming biofilms on different surfaces. Biofilms consist of a dense network of surface-attached yeast-like and filamentous cells surrounded by an extracellular matrix composed mainly of carbohydrates, proteins, phosphorus, uronic acid, and hexosamine [9,10]. These biofilm cells display a distinct characteristic regarding gene transcription and growth rate, as well as antifungal and host defense susceptibilities compared with planktonic cells [4,8]. Remarkably, this mode of growth provides fungal cells with resistance to several antifungal agents [8,9,10,11], and host defenses [8]. Thus, biofilm-associated infections are difficult to treat, and may explain the high mortality rates related to *C. tropicalis* candidemia [5].

In addition to biofilm formation, the antifungal resistance of planktonic cells has raised concerns in the treatment of infections caused by *C. tropicalis*. Due to its broad spectrum of action and relative safety, fluconazole (FLZ), an azole antifungal agent, has been used as a first-line drug in the treatment of several fungal infections [12]. However, the resulting selective pressure from this approach has contributed to the emergence of FLZ-resistant strains. An increase in the prevalence of azole- or multidrug-resistant (non-susceptible to ≥1 agent in ≥2 antifungal classes) [13] *C. tropicalis* isolates has been observed over the years [14,15,16,17].

Azoles are fungistatic compounds that inhibit the ergosterol biosynthesis pathway. The main target of FLZ is the cytochrome-P450-dependent lanosterol 14α-demethylase (encoded by the *ERG11* gene), an enzyme that converts lanosterol into ergosterol, resulting in the accumulation of 14-methylated sterol intermediates. These non-toxic intermediates are converted into the toxic sterol 14α-methylergosta-8,24(28)-dien-3β,6α-diol by the sterol Δ5,6-desaturase (encoded by the *ERG3* gene). Point mutations or the overexpression of *ERG11* [14,15,18,19,20] and/or *ERG3* [19,21] have been associated with the resistance of *C. tropicalis* to FLZ. Additionally, FLZ resistance can also arise due to the increased activity or overexpression of efflux pumps, which export antifungals from the inside to the outside of the cell [22]. In *C. tropicalis*, the efflux pumps named *Candida* Drug Resistance 1 (Cdr1) and 2 (Cdr2) [15,23,24], and Multi-Drug Resistance 1 (Mdr1), belonging to the ATP-Binding Cassette (ABC) superfamily and the Major Facilitator Superfamily (MFS) transporters [15,22,23], respectively, have been associated with azole resistance.

Given these growing concerns, the World Health Organization (WHO) has classified *C. tropicalis* as a high-priority fungal pathogen for developing new control strategies. Among the WHO recommendations for this issue, the assessment of the combined inhibitory effect between antifungals in vitro and in vivo could help to improve the current treatment regimens for *C. tropicalis* [6]. Owing to the great diversity of phytochemicals, plants have been shown to be promising sources of bioactive molecules for the discovery of new drugs or strategies for the treatment of fungal infections [25]. Previous studies have demonstrated the antifungal effect of geraniol (GER) against *Candida* species exhibiting different antifungal susceptibility profiles [26,27,28,29,30]. GER is a monoterpenoid extracted from the essential oil of several plants, whose chemical structure consists of two prenyl units linked in a head-to-tail arrangement and functionalized with a hydroxyl group [31]. GER has fungicidal properties, inhibiting the efflux pump activity of the ABC transporters in *C. albicans* [28], *C. glabrata* [29], and *C. auris* [30]. Additionally, GER exhibits a synergism with FLZ, reducing the minimum inhibitory concentration of the azole antifungal for *C. albicans* [27,28,32]. Although the antifungal activity of GER on *C. tropicalis* has been reported [33,34,35,36], the combined effect with FLZ on the planktonic cells and biofilms of azole-resistant strains has not been described yet. In this study, the antifungal effect of GER, alone and combined with FLZ, was evaluated against the planktonic and sessile (biofilm) cells of azole-resistant *C. tropicalis* in vitro and in vivo using the *Galleria mellonella* infection model. Furthermore, through an in silico and biochemical analysis, this study reports for the first time that GER can bind to the binding site of the *C. tropicalis* Cdr1 protein, inhibiting its activity and decreasing the minimum inhibitory concentration of FLZ.

## 2. Materials and Methods

### 2.1. Chemicals

Geraniol (GER, *trans*-3,7-dimethyl-2,6-octadien-1-ol, ≥98% purity), curcumin [CUR, (E,E)-1,7-bis(4-hydroxy-3-methoxyphenyl)-1,6-heptadiene-3,5-dione, ≥80% purity], Rhodamine 6G (R6G, ≥95% purity), 3-(4,5-dimethylthiazol-2-yl)-2,5-diphenyltetrazolium bromide (MTT), amphotericin B (AmB), penicillin, streptomycin, tylosin, dimethyl sulfoxide (DMSO), Tween^®^ 80, the Roswell Park Memorial Institute 1640 (RPMI) medium, and Dulbecco’s Modified Eagle medium (DMEM) were acquired from Sigma-Aldrich/Merck (São Paulo, Brazil). Fluconazole (FLZ, 99% purity) and itraconazole (ITR, 99% purity) were acquired from Fagron personalizing medicine (São Paulo, Brazil). TRIzol^™^, the BD Difco^™^ Yeast Extract Peptone Dextrose (YPD) broth, BD Difco^™^ CHROMagar *Candida*, and the LIVE/DEAD^™^ Yeast Viability Kit were acquired from ThermoFisher Scientific (São Paulo, Brazil). The QuantiNova SYBR^®^ Green RT-PCR kit and the RNeasy^®^ MinElute^®^ Cleanup kit were purchased from QIAGEN (São Paulo, Brazil). Sabouraud dextrose agar (SDA) and Sabouraud dextrose broth (SDB) were acquired from HiMedia (Maharashtra, India), and 3-(*N*-morpholino) propanesulfonic acid (MOPS) was acquired from INLAB (São Paulo, Brazil). Fetal bovine serum was acquired from Nova Biotecnologia (São Paulo, Brazil).

For all the antifungal assays, GER was dissolved in 1% DMSO and 0.5% Tween^®^ 80 to achieve a 4096 µg/mL stock solution. AmB, FLZ, and ITR were dissolved in DMSO to achieve a 4 mg/mL, 6 mg/mL, and 4 mg/mL stock solution, respectively. These stock solutions were maintained at −20 °C and were further diluted in the culture medium to obtain the concentrations used in each assay. DMSO and Tween^®^ 80 did not exceed 1% and 0.5%, respectively, in all assays.

### 2.2. Microorganisms

*C. tropicalis* ATCC 28707 (resistant to AmB) and three clinical *C. tropicalis* isolates recovered from urinary catheters were included in this study. These strains belong to the microbial collection of the Laboratory of Molecular Biology of Microorganisms at the State University of Londrina, Londrina, Paraná, Brazil.

*C. tropicalis* strains were cultivated on SDA at 37 °C for 24 h and stored at 4 °C. For the experiments, three colonies were transferred to SDB and incubated at 37 °C for 24 h. To prepare a standard fungal suspension, cells were harvested using centrifugation (10,000× *g* for 1 min) and resuspended in sterile 0.85% NaCl (saline) to achieve a turbidity equivalent to the 0.5 McFarland standard, using the DensiCHEK^™^ PLUS colorimeter (bioMérieux, Rio de Janeiro, Brazil). The standard fungal suspension, which corresponds to 1–2 × 10^6^ colony forming units (CFUs)/mL, was further diluted in the culture medium to achieve the inoculum used in the assays, except when specified.

### 2.3. Antifungal Effects on Planktonic Cells

#### 2.3.1. Minimum Inhibitory (MIC) and Minimum Fungicidal (MFC) Concentrations

The MIC values of GER, FLZ, ITR, and AmB were determined in U-bottom 96-well polystyrene plates (Techno Plastic Products, Trasadingen, Switzerland) using the broth microdilution assay, as recommended by the Clinical and Laboratory Standards Institute document M60 [37]. The stock solutions of GER, FLZ, ITR, and AmB were serially diluted in RPMI buffered with 0.165 M MOPS (RPMI-MOPS) to achieve assay concentrations ranging from 4 to 2048 μg/mL, 0.5 to 1024 μg/mL, 0.015 to 4.0 μg/mL, and 0.125 to 16 μg/mL, respectively. Wells containing the RPMI-MOPS medium supplemented with 1% DMSO and 0.5% Tween^®^ 80 with fungal cells served as the growth control, while the medium plus 1.0% DMSO and 0.5% Tween^®^ 80 served as the sterility control. The MIC was defined as the lowest concentration of the antifungal agent that inhibited visible fungal growth after 24 and 48 h of incubation at 37 °C compared with the growth control. The antifungal activity of GER was interpreted according to the following criteria: 0.005–500 μg/mL, strong activity; 600–1500 μg/mL, moderate activity; and >1500 μg/mL, inactive [38]. The susceptibility breakpoints for FLZ, ITR, and AmB were those recommended by the EUCAST [39].

To determine the MFC, aliquots (10 µL) from wells exhibiting no visible growth were homogenized, inoculated onto the SDA, and incubated at 37 °C for 24 and 48 h. The MFC was defined as the lowest concentration that inhibited 99.9% of the colony forming units (CFU) counts compared with the untreated control. The nature of the antifungal effect was interpreted according to the MFC/MIC ratio as follows: fungicidal, MFC/MIC = 1–4; fungistatic, MFC/MIC > 4 [40].

#### 2.3.2. Checkerboard Microdilution Assay

The antifungal effect of GER combined with FLZ was evaluated using the checkerboard microdilution method, as described by Longhi et al. [41]. Briefly, two-fold serial dilutions of GER (4–2048 µg/mL) and FLZ (0.031–8 µg/mL), and GER and ITR (0.015–4 µg/mL) in the RPMI-MPOS were added across the rows and columns of U-bottom 96-well microtiter plates. Fungal cells (1 × 10^3^ CFU/mL) were added to each well, and the plates were incubated at 37 °C for 48 h. The ratios of the MIC values obtained when the compounds were tested in combination and alone were used to determine the fractional inhibitory concentration (FIC) of each compound. The FIC index (FICI) was calculated as the sum of the FIC_GER_ and the FIC_FLZ_, and the values were interpreted as follows: synergistic (FICI ≤ 0.5), no interaction (0.5 < FICI < 4.0), or antagonistic (FICI > 4.0) [42].

#### 2.3.3. Time-Kill Assay

The rate of *C. tropicalis* killing by GER alone or combined with FLZ was analyzed using a time-kill assay [43]. Briefly, 1 mL of a fungal suspension (0.5– 2.5 × 10^5^ CFU/mL) was added to 9 mL of the RPMI-MOPS containing 0.5 × MIC, MIC, and 2 × MIC values of GER alone or the FLZ/GER combination at their synergistic concentrations. The cultures were incubated at 37 °C and, at specified time points (2, 4, 8, 12, and 24 h), a 10-µL aliquot was removed, diluted (1:10) in saline, and 100 µL of each dilution was inoculated onto the SDA. The CFU counts were carried out after incubation at 37 °C for 24 h. The averaged data were plotted as log10 CFU/mL versus time (h). The fungicidal effect of the compounds was defined as a 99.9% (3 log10) reduction in the CFU/mL compared with the starting inoculum [43].

### 2.4. Real-Time PCR

The relative mRNA levels of *ERG11*, *ERG3*, *CDR1*, and *MDR1* of *C. tropicalis* were determined using quantitative real-time PCR (qPCR). The nucleotide sequences of the *CDR1* and *ERG3* genes of *C. tropicalis* deposited in the GenBank/EMBL databases (available at http://www.ncbi.nlm.nih.gov, accessed on 10 January 2023) were used for the specific primer design. The sequences were analyzed using the BioEdit v.7.2.0 software. The primers were designed based on the consensus sequence, employing the OligoAnalyzer^™^ tool (http://www.idtdna.com, accessed on 10 January 2023). The primers targeting the *ERG11*, *MDR1*, and *ACT1* (encoding for actin) genes were those described by Bizerra et al. [10]. The *ACT1* gene was used as an internal control, and to normalize the expression levels of the target genes. The primer sequences, the annealing temperature, and the expected size of the amplicons for each gene are presented in Supplementary Table S1.

*C. tropicalis* planktonic cells were cultivated in a YPD medium containing 256 µg/mL of FLZ at 37 °C for 12 h. The total RNA was extracted using TRIzol^™^, as described by Silva et al. [20], and further purified using the RNeasy^®^ MinElute^®^ Cleanup kit following the manufacturer’s instructions. The qPCR assays were performed in a Rotor-Gene Q 5PlexHRM instrument (QIAGEN, Hilden, Germany), using total RNA (100 ng), 1 µM of each primer pair, and the recommended volume of the QuantiNova SYBR^®^ Green RT-PCR kit in a final volume of 20 µL. The cycling conditions were an initial step at 60 °C for 10 min and 95 °C for 2 min, followed by 35 cycles of denaturation at 95 °C for 30 s, annealing at 55 or 57 °C for 30 s, and extension at 72 °C for 30 s. A thermal dissociation curve analysis confirmed that qPCR generated specific amplicons for each primer pair. To determine the expression levels, the Ct (cycle threshold) values were normalized and analyzed using the REST2009 software (http://www.REST.de.com, accessed on 10 January 2023). The differences in mRNA levels were compared with the planktonic cells cultivated without FLZ (reference group).

### 2.5. Analyses of the Mode of Geraniol Action on Planktonic Cells

#### 2.5.1. Measurement of Cell Membrane Permeability

The release of the intracellular content (absorbing at 260/280 nm, such as nucleic acids and protein) from *C. tropicalis* was evaluated as described previously [44], with minor modifications. Briefly, an 18-h *C. tropicalis* culture was harvested using centrifugation (3000× *g*, 20 min, 4 °C), and 1 × 10^5^ CFU/mL were incubated in 20 mL 0.15 M phosphate-buffered saline, pH 7.2 (PBS) containing GER at 0.25 × MIC, 0.5 × MIC, and MIC at 37 °C at varying time intervals (0, 30, 60, and 120 min). After each incubation time, 2 mL of the cultures were taken and centrifuged at 3000× *g* for 20 min. For the evaluation of the intracellular content leakage, the OD at 260/280 nm of the supernatant was measured using a BioTek Synergy^™^ HT microtiter plate reader (Agilent, Santa Clara, CA, USA).

The effect of GER on the *C. tropicalis* cell membrane integrity was evaluated using the LIVE/DEAD^™^ Yeast Viability kit according to the manufacturer’s recommendations. *C. tropicalis* was treated with GER at MIC for 3 h. The treated and untreated fungal cells were incubated with FUN1^®^ and Calcofluor White^™^ M2R dyes and analyzed under a fluorescence microscope (OLYMPUS BX53, Center Valley, PA, USA) using fluorescein filters with excitation/emission wavelengths of 480/530 nm.

#### 2.5.2. Efflux Pump Inhibition

The efflux of rhodamine 6G (R6G) was determined as previously described [28,30,45]. Briefly, fungal cells (1 × 10^6^ cells/mL) from an 18-h incubation were inoculated into a YPD broth containing GER (128 µg/mL) and incubated at 37 °C for 5 h. Afterward, the cells were harvested using centrifugation (10,000× *g*, 1 min), washed twice with PBS, resuspended in the same buffer (1 × 10^8^ cells/mL), and de-energized for 1 h at 37 °C with constant agitation (150 rpm). After de-energization, the cells were centrifuged, washed (as described), and resuspended in PBS without glucose. Subsequently, R6G (10 µM) was added, and the cells were incubated under the same conditions. The cells were centrifuged after equilibration, resuspended in PBS supplemented with 2% glucose, and at specified time points (5–75 min), the energy-dependent R6G efflux was evaluated. For this, 1 mL of cell suspension was withdrawn, centrifuged, and the optical density (OD) of the supernatant was determined at 530 nm using a BioTek Synergy™ HT microtiter plate reader (Agilient, Santa Clara, CA, USA). CUR (37 µg/mL) [46] and glucose-free PBS were used as the inhibitors of the Cdr1 efflux pump activity and negative controls, respectively.

#### 2.5.3. Molecular Docking

##### Prediction of the 3D Structure of the *Candida tropicalis* Cdr1 Protein and Its Structural Validation

The sequence of the 1268 amino acids of the *C. tropicalis* drug resistance protein 1 (*Ct*Cdr1) was obtained from the NCBI reference sequence (XP_002545857.1) of *C. tropicalis* MYA-3404 (https://www.ncbi.nlm.nih.gov/protein/XP_002545857.1, accessed on 10 January 2023). The AlphaFold2 program was used to build three-dimensional (3D) models of the *Ct*Cdr1, with the help of the ColabFold v1.5.5 software (https://colab.research.google.com/github/sokrypton/ColabFold/blob/main/AlphaFold2.ipynb, accessed on 10 January 2023), which is freely available and uses a virtual machine to synthesize models [47,48]. The conformation of the *Ct*Cdr1 models predicted using AlphaFold2 was validated using the Ramachandran diagram. The analysis of the torsion angles phi (Φ) and psi (Ψ) was performed using the PROCHECK online server (https://saves.mbi.ucla.edu/, accessed on 10 January 2023) for the model conformation assessment. Furthermore, the model was also subjected to additional verification using the MolProbity online server (http://molprobity.biochem.duke.edu/index.php?MolProbSID=8hfh0bc9jbn3it1mchu67r8qi6&eventID=2, accessed on 10 January 2023) to confirm its structural integrity [49].

##### Binding Site Detection and Grid Box Formation

The binding site of the *Ct*Cdr1 was identified using the BIOVIA Discovery Studio (DS) Visualizer 21.1 software. Next, the grid mapping was performed to target the protein inhibitors, such as CUR and farnesol (FAR), as well as the GER ligand, in order to search for their regions of high affinity with the binding site of the *Ct*Cdr1 protein. The grid used for the *Ct*Cdr1 had dimensions of 40 × 40 × 40 grid points, with a spacing of 0.375 Å between the points. These points were centered on the coordinates of the ligand and *Ct*Cdr1 (−21.567; −0.843 and 20.058) [50].

##### Preparation of Ligands and the *Ct*Cdr1 Protein

Using Auto Dock Tools (ADT) version 1.5.6 (Scripps Research Institute, San Diego, CA, USA), hydrogen atoms with a polar nature were added to the predicted AlphaFold2 models of *Ct*Cdr1. Furthermore, Kollman charges were assigned to each atom, and nonpolar hydrogen atoms were incorporated into the protein structure. The resulting structures were saved in the PDBQT file format for further analysis using ADT [51]. Next, the 3D structures of the ligands CUR, FAR, and GER were prepared. These structures were obtained from PubChem NCBI (https://pubchem.ncbi.nlm.nih.gov/, accessed on 10 January 2023) in the ‘.sdf’ input format, pre-optimized with the Avogadro 1.2.0 software at pH 7.4 and using the force field MMFF94. Then, the optimization was completed in Mopac2012, using the PM7 level, generating outputs in the ‘.pdb’ format, and the AM1-BCC charges were then added using the UCSF Chimera 1.16 software, and the files were saved in the ‘.mol2’ format [52,53,54]. The ‘.mol2’ files were subsequently converted into the PDBQT format, using processes such as root detection, twist selection, and the definition of the number of twists, using ADT. Molecular docking simulations were performed using the Autodock 4.2 suite, employing the Lamarckian genetic algorithm [55].

### 2.6. Prolonged Exposure of the Fungal Cells to Geraniol

A single colony of *C. tropicalis* was inoculated into 5 mL of SDB, and incubated at 37 °C for 18 h. An aliquot (100 µL) of fungal cells (5 × 10^5^ cells/mL) was transferred to a fresh medium supplemented with GER and incubated at 37 °C for 24 h with constant agitation (130 rpm). Approximately 100 μL (5 × 10^5^ cells/mL) from each previous culture were transferred daily to a fresh medium containing increasing concentrations of GER, starting from 64 to 512 μg/mL. Fungal cells were subcultured three times in the presence of each concentration of GER. At 256, a 1.0 mL aliquot was used to assess the MIC of GER using the broth microdilution method, as described above. Additionally, every 3 days, 10 µL of the culture was withdrawn and inoculated onto a chromogenic medium to exclude contamination by other microbial species [23].

### 2.7. Antifungal Effect on the Sessile (Biofilms) Cells

#### 2.7.1. Adhesion on Polystyrene

The effect of the compounds on the adherence of *C. tropicalis* to a polystyrene surface was determined as previously described [56]. To prepare the inoculum, the fungal strains were grown on SDA at 37 °C for 24 h, and three colonies were transferred to SDB and incubated under the same conditions. The fungal cells were harvested using centrifugation (10,000× *g* for 1 min), washed three times with PBS (pH 7.2), and resuspended in the same buffer containing different concentrations of GER alone (128–1024 μg/mL) or combined with FLZ (0.5–4 μg/mL) to achieve a cell density of 1 × 10^7^ CFU/mL (estimated by direct counting using an Improved Double Neubauer hemocytometer). Afterward, 50 μL of each fungal cell suspension were added into the wells of a flat-bottom 96-well polystyrene plate (Techno Plastic Products, Trasadingen, Switzerland), and the systems were incubated at 37 °C for 90 min at 100 rpm. The wells were washed three times with PBS to remove the non-adherent cells, and the adherent cells were assessed by measuring the relative metabolic activity using the MTT reduction assay, as recommended by the manufacturer.

#### 2.7.2. Antibiofilm Activity

The growth kinetics of the *C. tropicalis* biofilms on the polystyrene surface were evaluated as described by Bizerra et al. [10], with minor modifications. The fungal inoculum was prepared as described above, except that the fungal strains were previously cultured in a YPD medium and then resuspended in RPMI-MOPS. Thereafter, 100 µL of the fungal suspension (1 × 10^6^ CFU/mL) were transferred to the flat-bottom 96-well polystyrene plate and incubated statically at 37 °C for 24, 48, 72, and 96 h. After each incubation period, the metabolic activity of the sessile cells was evaluated using the MTT, as above.

To determine the antibiofilm activity of the compounds, the biofilms of the *C. tropicalis* strains were formed as above. After incubation at 37 °C for 48 h, the biofilms were washed twice with PBS to remove the non-adherent cells. The RPMI-MOPS (100 µL) containing different concentrations of GER (32–2048 μg/mL) alone or combined with FLZ (0.25–4 µg/mL) was added, and the biofilms were incubated at 37 °C for an additional 24 h and then washed with PBS. The metabolic activity of the biofilms was analyzed using the MTT reduction assay as described above. The MIC of GER alone and combined with FLZ was determined as the lowest concentration capable of inhibiting 90% of the metabolic activity of the sessile cells (SMIC_90_) compared with the untreated controls.

#### 2.7.3. Scanning Electron Microscopy (SEM)

The morphological alterations in the biofilms treated with the compounds were analyzed using SEM. Strips of polystyrene (1.0 cm^2^) were immersed in wells of 24-well cell culture plates containing 1 mL of RPMI-MOPS inoculated with *C. tropicalis* (1 × 10^6^ CFU), and the system was incubated at 37 °C for 48 h. The biofilms were washed twice with PBS, and treated with GER alone (2 × MIC) or combined with FLZ (at the synergistic concentration for the sessile cells) for 24 h at 37 °C. The biofilms were then fixed with 2.5% (*v*/*v*) glutaraldehyde in a 0.1 M sodium cacodylate buffer (pH 7.2) at room temperature for 4 h, dehydrated with serial ethanol washes (30%, 50%, 70%, 90%, and 100%), critical point dried using CO_2_ (BAL-TEC, CPD 030), coated with gold, and observed under a FEI Quanta 200 scanning electron microscope (ThermoFisher Scientific, Hillsboro, OR, USA).

### 2.8. Toxicity Analyses

#### 2.8.1. In Silico Predictions of Pharmacokinetic and Toxicity Parameters (ADME-Tox)

The free online platform pkCSM (https://biosig.lab.uq.edu.au/pkcsm/prediction, accessed on 16 May 2023) was used to predict the Absorption, Distribution, Metabolism, and Excretion (ADME) properties of GER [57]. In addition, the toxicity and drug-likeness of GER were evaluated based on Lipinski’s rule of five [58], using the OSIRIS Property Explorer (https://www.organicchemistry.org/prog/peo/, accessed on 16 May 2023) and Molinspiration (https://www.molinspiration.com/, accessed on 16 May 2023) platforms [59].

#### 2.8.2. Toxicity to *Galleria mellonella* larvae

*G. mellonella* larvae (instar stage) were selected according to their similarity in size (200–250 mg) and without apparent color alterations. Groups of 10 larvae were used in all the assays and were placed separately in Petri dishes. The compound concentrations per kg of larvae were evaluated as follows: 256 and 512 µg/mL of GER; 256/1 µg/mL of the GER/FLZ combination. Groups of larvae inoculated with PBS, and PBS plus 1% DMSO plus 0.5% Tween^®^ 80 were used as the controls. For the assays, the larvae were previously cleaned with 70% ethanol, and 10 µL of PBS containing the compounds were injected into the larval hemocele via the last left proleg using a Hamilton syringe (Hamilton Company Inc., Reno, NV, USA). Then, the larvae were incubated at 37 °C in the dark, and survival was monitored every 12 h for up to 120 h. The number of dead larvae was assessed every 12 h through a visual analysis for the presence of dark spots on their bodies and a lack of movement in response to physical stimulation with forceps [60].

### 2.9. Antifungal Efficacy In Vivo

#### *Galleria mellonella* Infection and Antifungal Treatment

For *G. mellonella* infection, *C. tropicalis* ATCC 28707 was cultivated in SDB at 37 °C for 24 h. The fungal cells were centrifuged, washed with PBS, and resuspended in the same buffer supplemented with 100 µg/mL ampicillin to prevent bacterial contamination. To determine the lethal inoculum (LI), 1 × 10^4^, 1 × 10^5^, 1 × 10^6^, and 1 × 10^7^ fungal cells were inoculated into the hemocele in the last left proleg. Then, the larvae were incubated at 37 °C, and their survival was monitored for up to 120 h, as described above. The inoculum capable of killing 80% of larvae was defined as LI_80_ and used to evaluate the antifungal efficacy in vivo.

To evaluate the antifungal efficacy, the larvae were divided into six groups(i) non-infected and untreated; (ii) infected and untreated; (iii) infected and treated with PBS; (iv) infected and treated with 256 µg of GER/kg of larva; (v) infected and treated with 512 µg of GER/kg of larva; (vi) infected and treated with 256/1 µg/kg of GER/FLZ combination/kg of larva. The larvae were infected with LI_80_ via the last left proleg and incubated at 37 °C for 2 h. The treatment was carried out by inoculating the compounds via the last right proleg. The larvae were incubated at 37 °C and their survival was monitored as described above. At the end of the experiment, the hemolymph from the surviving larvae was collected to determine the fungal load. Subsequently, the collected samples were serially diluted (1:10) and inoculated onto the SDA, incubated at 37 °C, and the CFU counts were performed after 24 h.

### 2.10. Statistical Analyses

For the statistical analysis, the GraphPad Prism software version 8.0.0 (GraphPad Software, San Diego, CA, USA) was used. An ANOVA was employed to analyze the differences between groups, followed by Tukey’s post-test. A *p*-value < 0.05 was considered significant. All the experiments were performed in triplicate and repeated on two different occasions.

## 3. Results and Discussion

### 3.1. Candida tropicalis Strains Are Resistant to Azoles and the Resistance Mechanism May Be Associated with the Overexpression of ERG3 and CDR1 Genes

The reference strain *C. tropicalis* ATCC 28707 and the clinical isolates were resistant to FLZ, displaying MIC and MFC values higher than 256 µg/mL. Additionally, the clinical isolates were sensitive to amphotericin B (AmB) and resistant to ITR (Table 1). The MIC value of FLZ for the quality control strain *C. parapsilosis* ATCC 22019 was within the acceptable range [37,39]. To investigate the mechanism of azole resistance in the *C. tropicalis* strains of this study, the expression of the genes *ERG11*, *ERG3*, *MDR1,* and *CDR1* was analyzed using real-time PCR.

The analysis of the mRNA levels revealed that the *CDR1* gene was overexpressed in all the azole-resistant *C. tropicalis* strains incubated with 256 µg/mL (0.5 × MIC) of FLZ for 12 h compared with the untreated planktonic cells. The differences in the *CDR1* gene expression ranged from 3.14 ± 1.11- to 14.27 ± 2.45-fold. Furthermore, the overexpression of the *ERG3* gene was also observed for the reference ATCC 28707 strain and the clinical isolate CTR3, showing a difference in the expression of 3.48 ± 2.42-fold and 4.57 ± 2.45-fold, respectively, when compared with the cells incubated without FLZ (Supplementary Figure S1). The *ERG11* and *MDR1* expression were not upregulated in these strains. These results indicate an active role of the *CDR1* and *ERG3* gene in the azole resistance of the *C. tropicalis* strains analyzed in this study. Similarly, Rojas et al. [21] reported an overexpression of the *ERG3* gene in *C. tropicalis* exhibiting resistance or dose-dependent sensitivity to FLZ in the presence of this azole antifungal. In contrast to our results, previous studies reported the overexpression of the *MDR1*, *CDR2*, *CDR3,* and *ERG11* genes in azole-resistant *C. tropicalis* [18,23,24]. Other mechanisms, which were not investigated in this study, may also be associated with resistance to azoles in this fungal species.

### 3.2. Geraniol Exhibits Fungicidal Activity against the Planktonic Cells of Azole-Resistant Candida tropicalis, Impairing the Cell Membrane Permeability, and Prolonged Exposure to This Monoterpenoid Does Not Select for Resistant Mutants

Previous studies reporting the antifungal effect of GER against *Candida* species exhibiting different antifungal susceptibility profiles identified MIC/MFC values ranging from 16 to 1000 µg/mL [26,27,28,29,30,32,33,34,35,36]. In the present study, the antifungal effect of GER was analyzed against the azole-resistant *C. tropicalis* strains. The MIC and MFC values of GER, determined after a 24-h incubation, were 512 and 1024 µg/mL for all the strains, respectively (Table 1). A fungicidal effect was observed based on an MFC/MIC ratio of 2, according to the criteria of Balouri et al. [40]. Notably, no changes in the MIC and MFC values were observed after a 48-h incubation.

**Figure 1:**
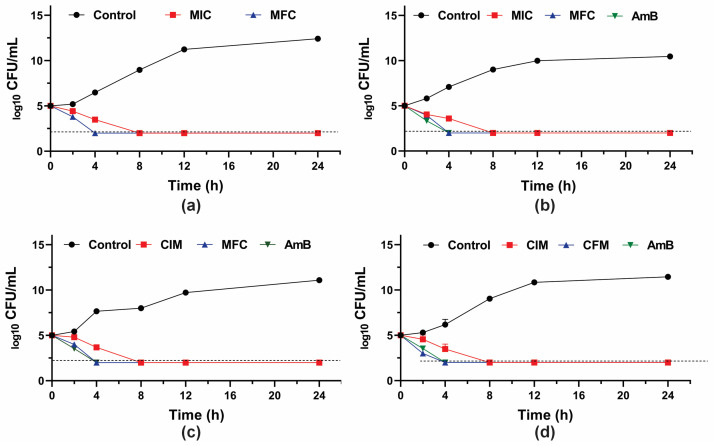
Antifungal activity of geraniol (GER) in *Candida tropicalis*. Time-kill kinetics of *C. tropicalis* ATCC 28707 (**a**); *C. tropicalis* CTR1 (**b**); *C. tropicalis* CTR2 (**c**); *C. tropicalis* CTR3 (**d**) incubated with the minimum inhibitory (MIC) and fungicidal (MFC) concentrations of GER. The log_10_ CFU/mL values are the mean and the standard deviation from three independent experiments. The dotted lines represent the 99.9% (3 log10) reduction in the CFU/mL counting. AmB: amphotericin B.

To further investigate the fungicidal activity of GER on *C. tropicalis*, the growth kinetics of the planktonic cells were monitored during 24 h in the presence of this compound (at the MIC and MFC values). A 3-log_10_ reduction in the CFU counts was observed after 8 h and 4 h of incubation with GER at the MIC and MFC, respectively (Figure 1), corroborating the fungicidal nature of this compound against these azole-resistant strains. The growth kinetics of the clinical strains exposed to GER at the MFC exhibited a similar pattern to that observed with AmB, a well-established fungicidal agent [61]. Similarly, a previous study reported a fungicidal effect of GER on *C. albicans* within the first hours (2–4 h) of incubation [26].

**Figure 2:**
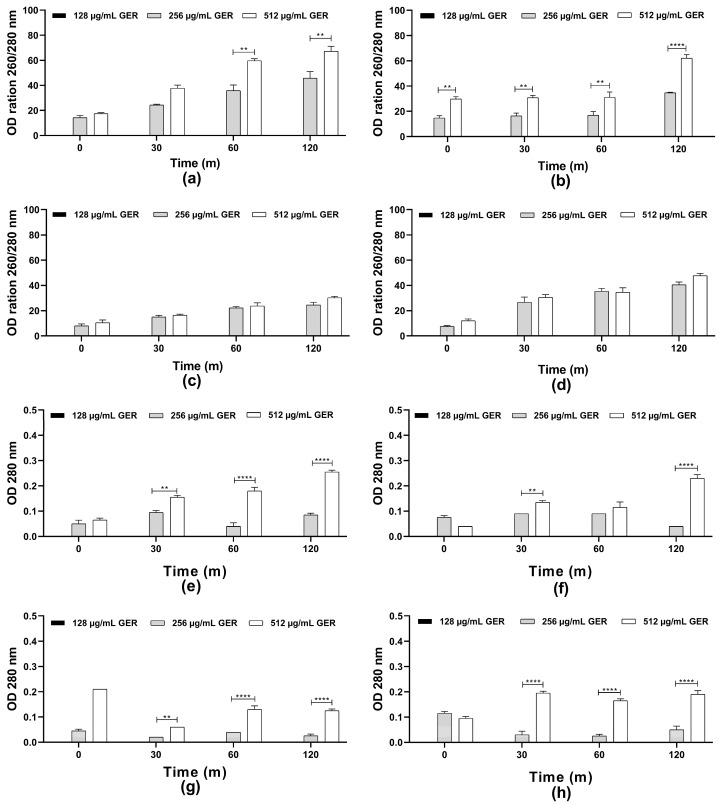
Plasma membrane integrity analysis of *Candida tropicalis* ATCC 2870. Planktonic cells were incubated with or without 0.25 × MIC, 0.5 × MIC, and MIC of geraniol. The intracellular content absorbing at 260/280 nm (**a**–**d**) or 280 nm (**e**–**h**) was determined after the specified time intervals. The values are the means and standard deviations from three independent experiments. (**a**,**e**) *C. tropicalis* ATCC 28707; (**b**,**f**) *C. tropicalis* CTR1; (**c**,**g**) *C. tropicalis* CTR2; (**d**,**h**) *C. tropicalis* CTR3. ** *p* <0.01, **** *p* < 0.0001 compared with the untreated fungal cells.

The scientific literature suggests that the fungicidal effect of terpenes and derivatives from different plant species on *C. albicans* may be due to the damage of the plasma membrane and the altered permeability [31]. For instance, the monoterpene isoespintanol, isolated from the leaves of *Oxandra xylopioides*, caused damage in the *C. tropicalis* plasma membrane, resulting in intracellular content (nucleic acid and proteins) leakage [44]. Furthermore, a dose-dependent decrease in the glucose-stimulated proton efflux mediated by H^+^-ATPase activity in *C. albicans*, *C. tropicalis*, and *C. glabrata* treated with GER has been observed previously [34,62]. Therefore, the possible effect of GER on the integrity of the *C. tropicalis* plasma membrane was analyzed by measuring the release of the intracellular content absorbing at 260/280 nm, including nucleic acid and proteins. Then, azole-resistant *C. tropicalis* strains were treated with GER (128, 256, and 512 µg/mL) and the OD_260 nm_/OD_280 nm_ values were monitored at 0, 30, 60, and 120 min. As shown in Figure 2a–h, at 128 µg/mL (0.25 × MIC), no leakage of the intracellular components absorbing at 260/280 nm was detected. In contrast, at 256 µg/mL (0.5 × MIC) and 512 µg/mL (MIC) a dose-dependent increase in these values was observed for all the fungal strains, indicating leakage of the intracellular components. This effect did not differ between both GER concentrations for the CTR2 and CTR3 strains (Figure 2c,d). Notably, the CTR1 strain exhibited a significant difference (*p* < 0.01) in leakage between the 256 and 512 µg/mL GER concentrations over the analysis period (Figure 2b). The reference strain displayed a significant difference in leakage between these GER concentrations only at the 60- and 120-min time points. For the components absorbing at 280 nm, a dose-dependent (256 and 512 µg/mL) leakage was observed for most of the *C. tropicalis* strains (Figure 2e–h). To further explore the effect of GER on the plasma membrane, *C. tropicalis* ATCC 28707 untreated and treated with GER at the MIC for 3 h were incubated with FUN1^®^ and Calcofluor White^™^ M2R fluorescent dyes for differential labeling. Red-orange fluorescent intravacuolar structures were observed within the cytoplasm of the untreated fungal cells, indicating metabolically active cells with intact cytoplasmic membranes (Supplementary Figure S2a,b). Conversely, cells treated with the GER MIC exhibited diffuse green-yellow fluorescence staining, indicative of impaired metabolic activity and damaged plasma membrane (Supplementary Figure S2c,d).

Overexposure to antifungal agents, resulting from long-term therapies, inappropriate use, and sometimes the indiscriminate application in human health, agriculture, and veterinary medicine, has contributed to the increase in fungal resistance, especially to azoles [4]. Therefore, the selection of GER-resistant *C. tropicalis* mutants was carried out by culturing yeast cells at increasing sub-MIC values for 30 days. After prolonged treatment with GER, no changes in fungal susceptibility were observed, as the MIC remained unchanged throughout the experiment, at 512 µg/mL. In summary, these results indicate that GER serves as a potential candidate in the development of novel control strategies targeting different *Candida* species.

### 3.3. Geraniol Can Inhibit the Efflux Pump Activity of Candida tropicalis by Binding to the Cdr1 Protein

**Figure 3:**
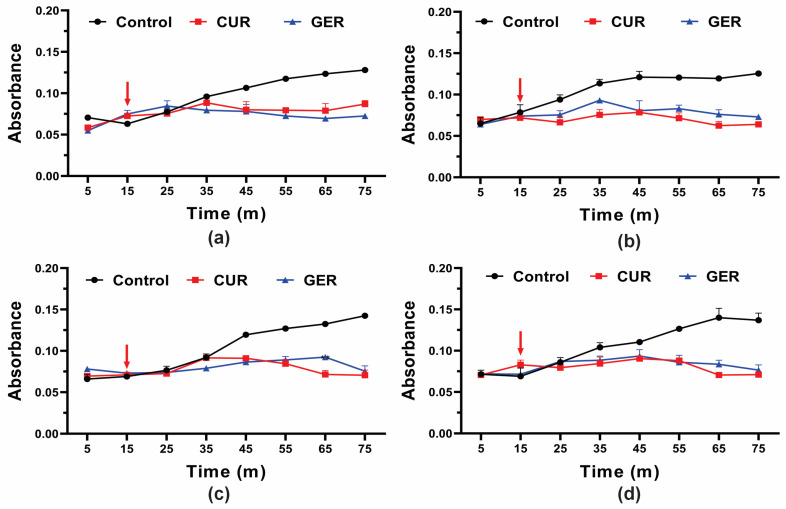
Effect of geraniol on Rhodamine 6G efflux by the planktonic cells of *Candida tropicalis*. Planktonic cells were incubated with 0.25 × MIC of geraniol for 5 h. The energy-dependent R6G efflux was initiated by adding 2% glucose (arrow) and quantified by measuring the absorbance of the supernatant at 530 nm. (**a**) *C. tropicalis* ATCC 28707; (**b**) *C. tropicalis* CTR1; (**c**) *C. tropicalis* CTR2; (**d**) *C. tropicalis* CTR3. The values are the means and standard deviations from three independent experiments. CUR: curcumin.

Previous studies have shown that GER abrogates the efflux pump activity of ABC transporters in *C. albicans* [28], *C. glabrata* [29], and *C. auris* [30]. Given the overexpression of the *CDR1* gene in azole-resistant *C. tropicalis* in this study, and to gain insights into the mode of action of GER, we evaluated its effect on efflux pump activity by monitoring the glucose-induced Rhodamine 6G (R6G) efflux. Since GER did not cause leakage of the intracellular contents (absorbing at OD_260/280nm_) at a concentration of 128 µg/mL, we evaluated whether this monoterpenoid could act as an efflux pump inhibitor in *C. tropicalis*. As shown in Figure 3, untreated control cells of all strains exhibited a gradual increase in R6G efflux upon glucose addition. Conversely, R6G efflux was inhibited in cells treated with a sub-inhibitory concentration of GER (128 µg/mL) and curcumin (CUR; 37 µg/mL), a known inhibitor of the *C. albicans* Cdr1p efflux pump [46].

**Figure 4:**
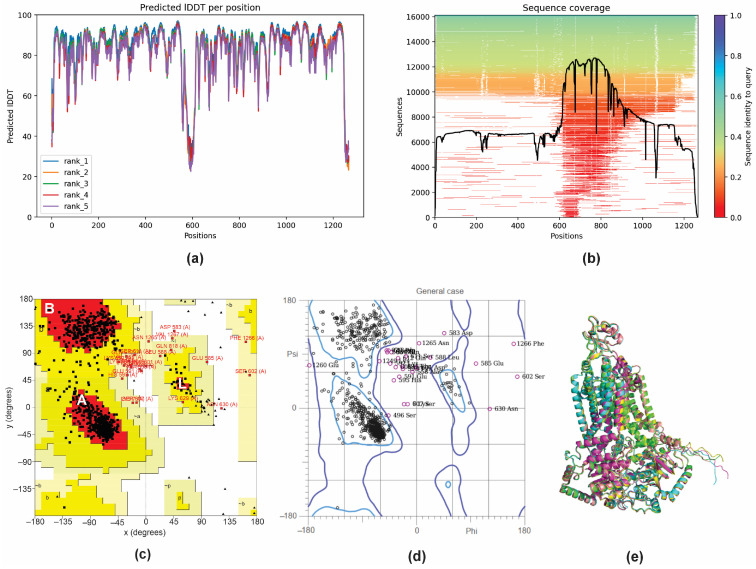
3D structure prediction and validation of *Candida tropicalis* resistance protein 1 (*Ct*Cdr1). Predicted pLDDT (**a**) and amino acid residue position coverage (**b**) of *Ct*Cdr1. Validation of the maximum score model using the PROCHECK Ramachandran plot (**c**) and the MolProbity Ramachandran plot (**d**). Alignment of the five 3D structures of *Ct*Cdr1 predicted using AlphaFold2 (**e**).

To further elucidate the observed inhibition of the efflux pump activity by GER, a molecular docking analysis was performed to investigate the potential interaction between the GER and Cdr1p of *C. tropicalis* (*Ct*Cdr1). Since there are no records of theoretical computational studies or the crystallographic structure of *Ct*Cdr1 in the Protein Data Bank (PDB), a model of this protein was predicted using AlphaFold2 through the ColabFold server. AlphaFold 2 is known for generating high-quality, three-dimensional (3D) protein structure predictions based on amino acid sequences [48]. The *CDR1* gene nucleotide sequence was obtained from the genome of *C. tropicalis* MYA-3404. This gene encodes a polypeptide with 1268 amino acid residues, which was then used to predict its 3D structure. Based on the α-carbon torsion angles phi (Φ) and psi (Ψ), several predictions of *Ct*Cdr1 were made and ranked using the predicted local distance difference test (pLDDT) score. The pLDDT score calculates the difference between the actual and predicted interatomic distances in a protein structure, with values greater than 70% representing the best-fitting model [63]. In this study, the top-ranked showed a pLDDT score greater than 84%, indicating that *Ct*Cdr1 was adequately modeled and suitable for subsequent applications (Figure 4a). The sequence coverage of the template was based on 14,000–16,000 homologous sequences to produce the model, and the queried sequences reached more than 70% identity. The regions without coverage (gaps) did not affect the binding site region of the protein (Figure 4b). To ensure the reliability of the best-predicted *Ct*Cdr1 model, we obtained Ramachandran plots independently from the PROCHECK and MolProbity servers. The PROCHECK Ramachandran plots showed that more than 88.4% of the amino acid residues of *Ct*Cdr1 were in the most favorable regions (A, B, and L), indicating a high-quality model (Figure 4c). Moreover, the distribution of the residues within the allowed regions indicates a consistent and well-folded dihedral conformation of the protein [49]. Similarly, the MolProbity Ramachandran plots showed that 96.9% of *Ct*Cdr1 residues were within the allowed regions, further confirming the quality of the predicted model (Figure 4d). Finally, the alignment of the top five predicted models showed a root mean square deviation (RMSD) of 0.452 Å (Figure 4e), supporting the reliability of the selected model.

Molecular docking with the AutoDock4.2 software was used to predict the binding mode of GER within the binding site of *Ct*Cdr1. For comparison, we also analyzed farnesol (FAR) and curcumin (CUR), which are known selective inhibitors of the ABC superfamily efflux pumps [46,64]. The intermolecular interaction analyses and figures were generated using the BIOVIA Discovery Studio Visualizer program. We chose the 3D conformation with the minimum binding energy (ΔG) values and inhibitory constant (Ki) for each molecule for further analysis, and the results are presented in Table 2. The minimum Gibbs free energy associated with forming a complete ligand-receptor (ΔG) value of −5.61 kcal/mol indicates that GER binds to the binding site of *Ct*Cdr1. Extra information about the binding energy and specific interaction of *Ct*Cdr1 with GER, CUR, and FAR is shown in Supplementary Table S3.

**Figure 5:**
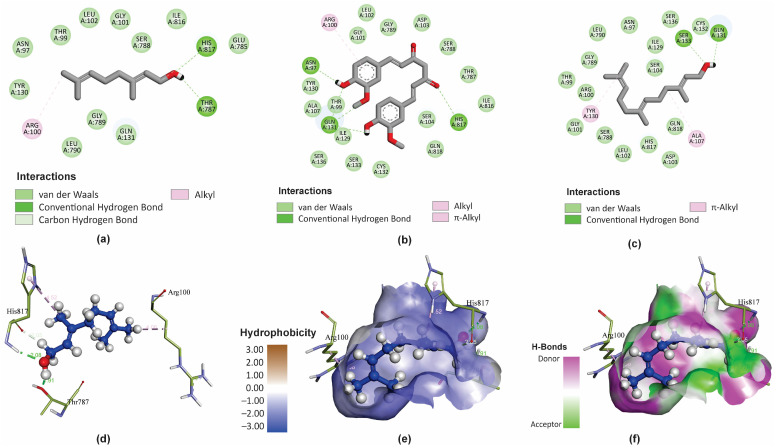
Molecular interactions of geraniol (GER), curcumin (CUR), and farnesol (FAR) with *Candida tropicalis* resistance protein 1 (*Ct*Cdr1). The main types of binding of GER (**a**), CUR (**b**), and FAR (**c**) to the binding site of the *Ct*Cdr1p) in 2D. The 3D distribution and chemical binding distances of GER (blue) with the amino acids (green) of the *Ct*Cdr1p binding site (**d**). 3D surface models showing the regions of the ligand (GER) with higher or lower degrees of hydrophobicity and the hydrogen donor and acceptor sites (**e**,**f**).

GER is a small molecule mostly exhibiting lipophilic physicochemical properties, with only one polar functional group (OH) (Figure 5a). However, GER displays bioactivity against *C. tropicalis* by interacting with important residues within the binding site of the *Ct*Cdr1 protein, possibly inhibiting its activity. It is also worth highlighting the low hydrophobicity of the binding site of *Ct*Cdr1, which justifies the lower affinity of GER for this pocket, composed mainly of hydrophilic residues. Actually, the amphipathic character of GER facilitates interactions with these hydrophobic regions of the *Ct*Cdr1 binding site, forming hydrogen bonds with His817 and Thr787 residues.

Additionally, the C1 and 7-methyl group of GER can also interact with the His817 and Arg100 residues, respectively, through carbon–hydrogen bonds (Figure 5a). Conversely, CUR forms hydrogen bonds with Asn97, Gln131, and His817 (Figure 5b), while FAR interacts with the Ser133 and Gln131 residues (Figure 5c) within the *Ct*Cdr1 binding site. In addition, the *Ct*Cdr1 interacts with GER, CUR, and FAR, through alkyl, π-alkyl, hydrogen–carbon bonds, and van der Waals interactions. Moreover, amide-π stacked bonds were observed between *Ct*Cdr1 and CUR and FAR. Similarly, a ΔG of -8.8 kcal/mol was observed for GER as a ligand of the *C. albicans* Cdr1 protein (*Ca*Cdr1), displaying hydrogen bond interactions with Pro1061 and Ser1062, in addition to hydrophobic interactions with several amino acid residues in its binding site [28]. Collectively, these results support that GER binds to the binding site of the *Candida* spp. Cdr1 proteins, contributing to its antifungal effect.

### 3.4. Geraniol Displays a Synergistic Interaction with Fluconazole against Planktonic Cells of Azole-Resistant Candida tropicalis

The antimicrobial combination therapy has been widely used to treat various infectious diseases [65], including those caused by fungal species [66]. This strategy aims to improve the effectiveness of drugs, as well as reduce the dosage and their adverse effects. Furthermore, it is an important tool for tackling antimicrobial resistance [60,65].

Considering the inhibitory effect of GER on the activity of efflux pumps, we evaluated its effect on FLZ susceptibility in azole-resistant *C. tropicalis* strains overexpressing the *CDR1* gene. The antifungal effect of GER combined with FLZ or ITR was investigated using the checkerboard assay. The fractional inhibitory concentration index (FICI) of GER (256 µg/mL) with FLZ (1 µg/mL) was 0.50 for all the *C. tropicalis* strains, indicating a synergistic interaction according to the interpretation criteria suggested elsewhere [42].

**Figure 6:**
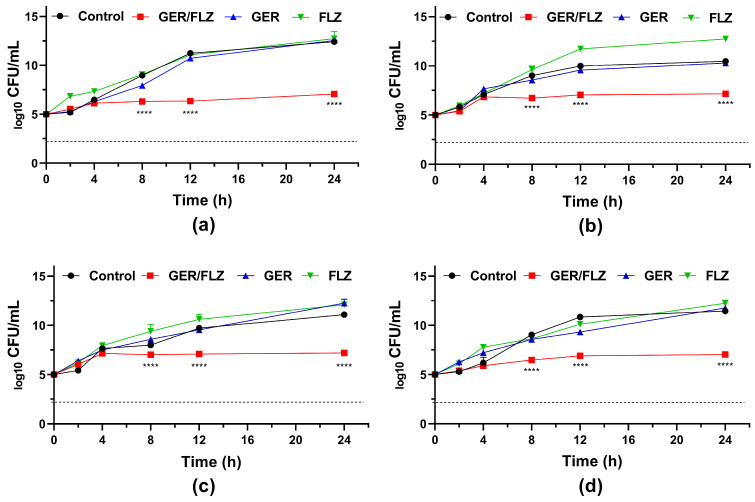
Antifungal activity of geraniol (GER) alone and combined with fluconazole (FLZ) against the planktonic cells of *Candida tropicalis*. Time-kill kinetics of *C. tropicalis* ATCC 28707 (**a**); *C. tropicalis* CTR1 (**b**); *C. tropicalis* CTR2 (**c**); *C. tropicalis* CTR3 (**d**) incubated with GER (256 µg/mL) and FLZ (1 µg/mL) alone or in combination (GER/FLZ, 256/1 µg/mL). The dotted lines represent the 99.9% (3 log10) reduction in the CFU/mL counting. The log_10_ CFU/mL values are the mean and the standard deviation from three independent experiments. **** *p* < 0.0001 compared with the untreated fungal cells.

Although the synergistic concentration of GER/FLZ presented a fungistatic effect (Figure 6), the simultaneous addition of GER with FLZ caused at least a 256-fold reduction in the MIC values of the FLZ agent for all the *C. tropicalis* strains analyzed in this study. Similarly, a synergistic interaction between GER and ITR was observed, and an FICI value of 0.26 was identified for the CTR1 strain, and 0.27 for the other *C. tropicalis* strains. At least a 64-fold reduction in the MIC values of ITR was observed (Supplementary Table S2).

The antifungal effect of the combination is supported by the fungal growth in the presence of GER alone and FLZ alone at concentrations that showed synergism. As shown in Figure 6, the growth kinetics patterns in the presence of this monoterpenoid (256 µg/mL) and FLZ (1 µg/mL) were similar to the untreated control for all the *C. tropicalis* strains analyzed. Similarly, Cardoso et al. [27] and Singh et al. [28] reported a synergistic antifungal interaction between GER and FLZ against the planktonic cells of FLZ-resistant *C. albicans*. However, both studies did not evaluate the nature of this synergistic combination.

### 3.5. Geraniol Alone or Combined with Fluconazole Inhibits Azole-Resistant Candida tropicalis Biofilms Formed on Abiotic Surfaces

The inhibitory effect of terpenoids from plants on adhesion and biofilm formation by *C. albicans* has been reported previously [56]. Specifically, GER inhibited the biofilms formed on different abiotic surfaces by *C. albicans* [27,28], *C. tropicalis* [33,36], *C. glabrata* [29], and *C. auris* [30]. Therefore, we evaluated the effect of GER alone or combined with FLZ on the adhesion and biofilms of azole-resistant *C. tropicalis*.

All the *C. tropicalis* strains used in this study were capable of forming biofilms on the polystyrene surface, exhibiting similar growth kinetics (Supplementary Figure S3). During the first 24 h, there was a gradual increase in the metabolic activity of sessile cells, which remained elevated until 48 h. Afterward, the metabolic activity gradually decreased. Previous studies showed that the initial phase of *C. tropicalis* biofilm formation begins with the adherence of yeast cells to the substrate, followed by the formation of yeast microcolonies, pseudohyphae, and hyphae. Mature biofilms consist of a dense network of yeast and filamentous cells embedded within exopolymeric substances [4,9,10,33].

**Figure 7:**
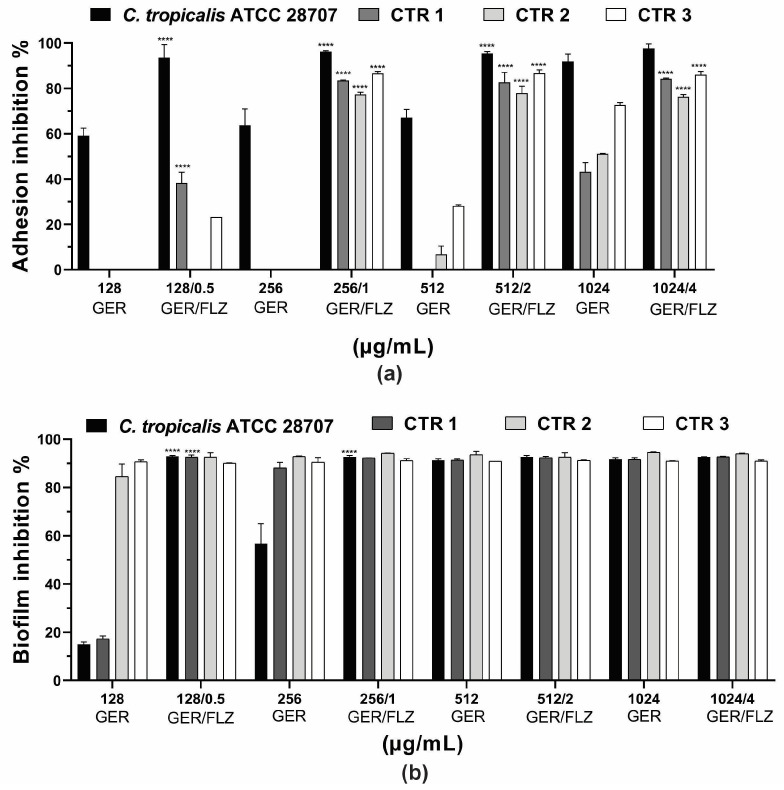
Effect of geraniol (GER) alone or combined with fluconazole (GER/FLZ) on *Candida tropicalis* adhesion (**a**) and 48-h biofilms (**b**) formed on polystyrene surface. The effect of GER alone or the GER/FLZ combination was evaluated using colony forming units counting, and the values were converted into percentages, considering the untreated groups as controls. Values are the mean and standard deviation from three independent experiments. **** *p* < 0.0001 compared with untreated fungal cells.

Microbial adhesion to surfaces is an important event in the pathogenesis of infections. It can trigger the invasion of host tissues, as well as the formation of biofilms on biotic or abiotic surfaces [4]. Previous studies have reported the inhibitory effect of GER at sub-inhibitory concentrations on *C. albicans* adherence to polystyrene (50% reduction) [62] and human buccal epithelial cells [28]. In this study, GER alone (0.25 × MIC, 0.5 × MIC, MIC, 2 × MIC) inhibited the adhesion of the reference ATCC strain at all the concentrations analyzed, causing a 60.1–91.9% reduction in the cells adhering to the polystyrene surface compared with the untreated control. In contrast, the adhesion of the *C. tropicalis* CTR1, CTR2, and CTR3 strains was inhibited at 1024 µg/mL, resulting in a 43.2–72.6% reduction in the adhered cells. GER at 512 µg/mL also inhibited the adhesion of CTR2 (6.74%) and CTR3 (28.1%). Overall, the combination of GER and FLZ (at 0.5 × MIC, MIC, 2 × MIC, and 4 × MIC of compound combination) significantly reduced the percentage of the adhered cells on polystyrene, ranging from 77.3% to 97.7%. Notably, at the synergistic concentrations of GER/FLZ determined for the planktonic cells (256/1 µg/mL), reductions in adhered cells of 96.2%, 83.4%, 77.4%, and 86.6% were observed for the reference, CTR1, CTR2 and CTR3 strains, respectively, and these results did not differ from those obtained with the other compound combinations (Figure 7a).

Next, we evaluated the effect of the compounds, alone or combined, on 48-h biofilms formed on polystyrene surfaces. A dose-dependent inhibitory effect of GER on 48-h biofilms was observed for all the *C. tropicalis* strains, and the percentage of the reduction in the metabolic activity of sessile cells ranged from 15% to 90.9% (Figure 7b). GER also inhibited the formation of *C. albicans* biofilms on polystyrene [62] and silicone [28] surfaces at sub-inhibitory concentrations (135 µg/mL). Moreover, Souza et al. [33] reported an antibiofilm effect of GER (500 µg/mL) on 24-h biofilms of *C. tropicalis* formed on plastic surface, with approximately 50% decrease in metabolic activity. Notably, there was no significant variation in the antibiofilm effect among all the GER/FLZ combinations for the fungal strains analyzed in this study, demonstrating a reduction in the metabolic activity of sessile cells greater than 90% (Figure 7b).

**Figure 8:**
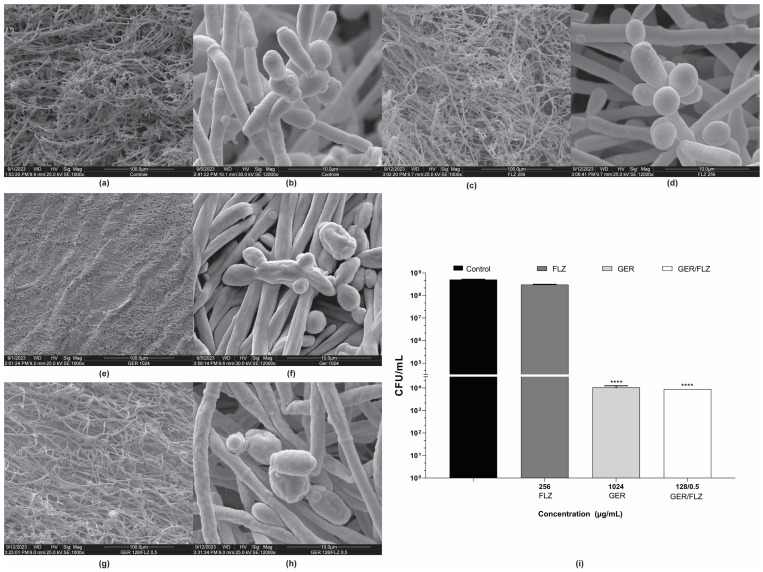
Effect of geraniol (GER) alone or combined with fluconazole (FLZ) on the morphology and ultrastructure of *Candida tropicalis* ATCC 28707 biofilms. Scanning electron microscopy (SEM) images of biofilms on polystyrene during 48 h of incubation. (**a**,**b**) Untreated control; (**c**,**d**) treated with 128 µg/mL FLZ; (**e**,**f**) treated with 1024 µg/mL GER; (**g**,**h**) treated with 128/0.5 µg/mL GER/FLZ. Viability of sessile cells after treatment with GER alone or GER/FLZ combination was evaluated using colony forming units counting (**i**). Values are the mean and standard deviation from three independent experiments. **** *p* < 0.0001.

Untreated and treated 48-h biofilms of *C. tropicalis* ATCC 28707 formed on a polystyrene surface were also analyzed using scanning electron microscopy (SEM). The untreated biofilm consisted of a dense and heterogeneous network of cells, mainly filamentous forms, with intense budding. The presence of water channels in the biofilm architecture was also observed (Figure 8a,b). The biofilm treated with FLZ (256 µg/mL) (Figure 8c,d) exhibited a similar architecture to the untreated control. In contrast, treatment with GER (1024 µg/mL) caused significant changes in the biofilm architecture, decreasing its stacking layers, and wilted cells were also observed (Figure 8e,f). Similar changes were also observed in the 24-h biofilms of *C. glabrata* after treatment with 100 µg/mL GER [29]. The GER/FLZ combination (128/0.5 µg/mL) caused morphological changes, with the surface cells of the biofilm exhibiting a rough and wrinkled texture (Figure 8g,h).

To support the fungicidal activity of GER alone or combined with FLZ on biofilms, the viability of the sessile cells of *C. tropicalis* 28707 was evaluated using CFU counts after treatment with GER (1024 µg/mL) or GER/FLZ (128/0.5 µg/mL). There was no statistically significant difference (*p* > 0.05) in CFU counts between the biofilms treated with GER alone or combined with FLZ. Moreover, a 3-log CFU reduction in CFU was observed, compared with the untreated control (Figure 8i).

Interestingly, the concentrations of the GER/FLZ combination for the antibiofilm activity were slightly lower when compared with their antifungal effect on planktonic cells (128/0.5 µg/mL for sessile cells versus 256/1 µg/mL for planktonic cells). Considering that *C. tropicalis* is known as one of the main biofilm-producing *Candida* species [4], these results highlight the potential of the GER/FLZ combination in controlling infections associated with biofilms.

### 3.6. In Silico Analyses Show That Geraniol Presents the Pharmacokinetic and Drug-Likeness Properties of a Safe Candidate for the Development of a New Drug

Defining the pharmacokinetic and toxicity profile of a new drug candidate is essential to minimize the risk of therapeutic failures or adverse effects during clinical trials [67,68]. Along with the pharmacological effects, the pharmacokinetic and toxicity characteristics of a compound define its absorption, distribution, metabolism, excretion, and toxicity properties, commonly known as the ADME-Tox properties. This information is crucial in the drug development process to ensure the safety and efficacy of the drug.

Most drug research studies aim to develop orally bioactive molecules due to the preferred characteristics of oral medication, which is non-invasive and conducive to patient compliance [69]. Lipinski’s rules are commonly used to determine the oral bioavailability of compounds [58]. These rules suggest that compounds with a molecular weight (MWT) exceeding 500 g/mol, a calculated Log P (cLogP) greater than 5, more than ten hydrogen bond acceptors (HBA), and more than five hydrogen bond donor groups (HBD), poor oral absorption and membrane permeation are more likely. The ADME-Tox properties of GER were predicted in silico, and the results are shown in Table 3. These findings indicate that GER does not violate Lipinski’s rules, suggesting it has drug-like properties and potential efficacy as an oral drug. Additionally, a topological polar surface area (TPSA) of 10.23 Å supports the notion that GER has a good oral bioavailability [70]. In fact, GER was designated as “generally recognized as safe” (GRAS) by the Flavoring Extract Manufacturers’ Association (FEMA) in 1965 [71] and was approved for food use by the United States Food & Drug Administration (FDA) in 2023 [72].

Furthermore, it was observed that GER has a relatively low skin permeability (logKp > −2.5) and easily crosses the blood–brain barrier (BHC) (logBB > 0.3) and the central nervous system (CNS) (logPS > −2.0). However, its volume of distribution at a steady state (VDss) is low (logVDss > 0.45), but it can present around a 56% free fraction. Additionally, GER showed no mutagenic or hepatotoxic effects, and does not seem to interfere with the main isoenzymes of the cytochrome P450 (CYP450) complex. Finally, its clearance is mainly attributed to renal excretion (Table 4).

The data in the literature support the notion that the antifungal concentrations of GER combined with FLZ against azole-resistant *C. tropicalis*, as described in the present study, are nontoxic to mammalian cells in vitro. For instance: (i) the cytotoxic concentrations of GER capable of inhibiting 50% of the metabolic activity in the RAW macrophage cell line [27] and the normal human lung fibroblasts (GM07492A) cells [33] were 380 µg/mL and 666.36 ± 28.39 μg/mL, respectively, which is higher than the concentrations showing synergistic antifungal effects (256/1 μg/mL for the planktonic cells and 128/0.5 μg/mL for the sessile cells); (ii) GER (225 µg/mL) caused minimal hemolysis in human erythrocytes [28]; (iii) GER at 800 µg/mL increased the chromosome damage in Chinese hamster lung fibroblast cells (V79 cells) [35], which is higher than the antifungal concentrations against *C. tropicalis*. However, GER at 31.3 μg/mL exhibited embryotoxic effects in a zebrafish model [35]. Further in vivo studies in mammals should be conducted to strengthen the application of GER as an antifungal agent that can be used safely in pharmaceutical formulations.

### 3.7. Geraniol, Alone or Combined with Fluconazole, Does Not Cause Toxicity to the Galleria mellonella Model, and Reduces Mortality in Larvae Infected with Azole-Resistant Candida tropicalis

*G. mellonella* (Lepidoptera, *Pyralidae*) larvae were used in this study to evaluate the efficacy of GER alone or combined with FLZ therapies through a survival assay in vivo. Due to characteristics such as small size, rapid growth, a short life cycle, and the ease of use in laboratory conditions, at different temperatures, including the human body temperature (37 °C), the larvae of this insect have been used as an alternative model to the use of mammalian models in research. Furthermore, their immune system shares many features with the human innate defense, enabling the development of immune responses against different microbial infections [73,74]. Indeed, the larvae of *G. mellonella* have been used to study the fungal virulence and pathogenesis [75], and to evaluate the toxicity and efficacy of antifungals against several fungal species [60,76].

**Figure 9:**
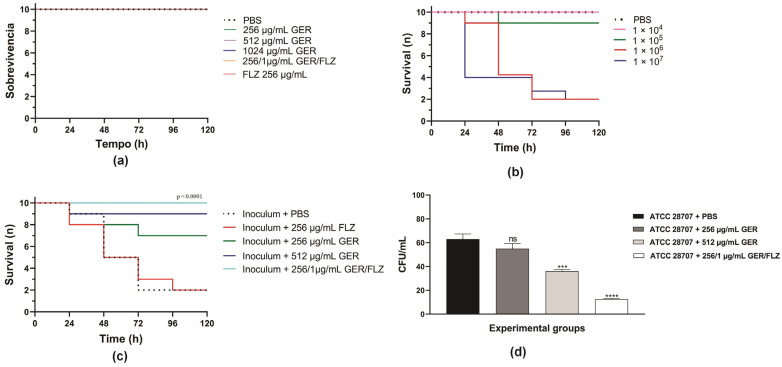
Kaplan–Meier plots of survival curves of *Galleria mellonella* larvae. (**a**) The larvae were inoculated with geraniol (GER), fluconazole (FLZ), and the GER/FLZ combination for the compounds toxicity analysis. (**b**) The larvae were infected with different *C. tropicalis* ATCC 28707 cell densities for determination of the lethal inoculum. (**c**) The larvae were infected with fungal cells (1 × 10^6^) and treated with (GER), fluconazole (FLZ), and the GER/FLZ combination. All the groups were compared with infected and untreated larvae. (**d**) Fungal load in the hemolymph of the larvae untreated and treated with the compounds determined using the colony forming unit (CFU) counts. The analysis of the *G. mellonella* survival data was performed using the log-rank (Mantel–Cox) of one representative experiment. The asterisks indicate a significant reduction in the fungal load of the infected treated group compared with the infected untreated group (ns, not significant; *** *p* < 0.001; **** *p* < 0.0001).

Initially, we evaluated the toxicity of GER alone (at 0.5 × MIC and MIC/kg of larva) or combined with FLZ (at a synergistic concentration/kg of larva) on *G. mellonella* larvae. A 100% survival rate was observed in the PBS-treated control group and all the groups treated with GER and FLZ after 5 days, indicating that these compounds do not cause toxicity at the tested concentrations (Figure 9a). Next, we determined the lethal inoculum of *C. tropicalis* ATCC 28707 on *G. mellonella* larvae, and the results are shown in Figure 9b. A gradual reduction in larval survival was observed with inocula of 1 × 10^6^ and 1 × 10^7^ CFUs of *C. tropicalis*, reaching 50% mortality after 48 h and 24 h post-infection. However, after 72 h post-infection, 80% mortality was observed for both the yeast inocula. Conversely, for 1 × 10^4^ or 1 × 10^5^ CFUs, survival rates of 100% and 90% were observed after 72 h.

Based on these results, the therapeutic efficacy of these compounds was evaluated in *G. mellonella* infected with *C. tropicalis* ATCC 28707 (1 × 10^6^ CFU), and the treatments were administered at 0.5 × MIC (256 µg/mL) and MIC (512 µg/mL) of GER, as well as at synergistic GER/FLZ concentrations (256/1 µg/mL). As shown in Figure 9c, treatment with GER increased the survival rate of larvae infected with azole-resistant *C. tropicalis*, resulting in 30% and 10% mortality rates after the administration of 256 and 512 µg/mL/kg of larvae, respectively. GER also exhibited antifungal activity at a sub-inhibitory concentration in *Caenorhabditis elegans* infected with FLZ-resistant *C. albicans* SC5314-Act1p-GFP (135 µg/mL) [28] and *C. auris* CBS10913T (65 µg/mL) [30], enhancing the larval survival after 3 and 7 days post-infection, respectively.

Treatment with GER/FLZ at the synergistic concentration resulted in a larval survival rate of 100% at the end of the experiment. Additionally, the fungal load in the hemolymph of all the surviving larvae was determined using the CFU counts. There was a significant reduction in the fungal load in infected larvae treated with the GER/FLZ combination compared with the other treatment groups. GER at 512 µg/mL also caused a significant reduction in the fungal load in larvae compared with those treated with 256 µg/mL and untreated larvae (Figure 9d). Similarly, GER/FLZ at a synergistic concentration increased survival and reduced fungal load in the hemolymph of larvae infected with 1 × 10^7^ *C. tropicalis* CFUs (Supplementary Figure S4). Altogether, our results indicate that GER has potential as an adjuvant antifungal in the treatment of infections caused by azole-resistant *C. tropicalis*.

The limitations of this study include the number of clinical isolates tested that do not represent all the antimicrobial susceptibility profiles of *C. tropicalis*, predominantly in in vitro testing conditions, and the utilization of an insect model in in vivo assays. Further studies are needed to evaluate the therapeutic potential and safety of GER alone or combined with FLZ in the treatment of *C. tropicalis* infections in mammals. Despite these limitations, our findings support GER as an antifungal agent and may provide new insights to improve therapeutic strategies for FLZ-resistant *C. tropicalis* infections.

## 4. Conclusions

The present study reports the antifungal activity of geraniol (GER), alone and combined with fluconazole (FLZ), against planktonic and sessile (biofilm) cells of azole-resistant *C. tropicalis*. The key findings include: (i) GER exhibits fungicidal activity against planktonic and sessile cells by impairing the cell membrane permeability; (ii) prolonged exposure to GER does not induce the development of GER-resistant *C. tropicalis*; (iii) GER exhibits synergism with FLZ and this effect may be attributed to the inhibition of the *Ct*Cdr1 efflux pump activity due to the binding of GER to this protein; (iv) the GER/FLZ combination inhibits the adhesion of yeast cell and pre-formed biofilms on the polystyrene surface; (v) the GER/FLZ combination does not cause toxicity and increases the survival of *G. mellonella* larvae infected with azole-resistant *C. tropicalis*. These results indicate that the combination of GER and FLZ may be a promising strategy to control azole-resistant *C. tropicalis* infections.

## Figures and Tables

**Table 1:** Antifungal activity of geraniol and commercial antifungals on the planktonic cells of *Candida tropicalis*.

*Candida tropicalis*	FLZ	ITR	AmB	GER	
	MIC/MFC (µg/mL)	MIC/MFC (µg/mL)	MIC/MFC (µg/mL)	MIC/MFC (µg/mL)	MFC/MIC *
*C. tropicalis* ATCC 28707	>256/>256	>0.5/>0.5	-	512/1024	2
CTR 1	>256/>256	>0.5/>0.5	1/1	512/1024	2
CTR 2	>256/>256	>0.5/>0.5	1/1	512/1024	2
CTR 3	>256/>256	>0.5/>0.5	0.5/0.5	512/1024	2

FLZ: Fluconazole; GER: Geraniol; AmB: Amphotericin B; ITR: Itraconazole; MIC: Minimum inhibitory concentration; MFC: Minimum fungicidal concentration. The susceptibility breakpoints for FLZ, ITR, and AmB were those recommended by the EUCAST [39]. * Reference values: 1–4 fungicidal and >4 fungistatic [40].

**Table 2:** Binding energy of the binding site of *Ct*Cdr1 with curcumin, farnesol, and geraniol.

	Enzyme	^a^ CUR		^b^ FAR		^c^ GER	
		^d^ Δ*G* (kcal/mol)	^e^ K_i_	^d^Δ*G* (kcal/mol)	^e^ K_i_	^d^ Δ*G* (kcal/mol)	^e^ K_i_
*C. tropicalis*	Cdr1	−9.55	99.73 nM	−7.00	7.41 µM	−5.61	77.56 µM

^a^ Curcumin; ^b^ Farnesol; ^c^ Geraniol; ^d^ Binding free energy; and ^e^ Calculated inhibition constant.

**Table 3:** In silico toxicological properties of geraniol and Lipinski parameters, Osiris property explorer.

Toxicological Properties		Lipinski’s Parameters	
Mutagenic	N	Molecular weight (g/mol)	154.25
Tumorigenic	N	TPSA	20.23
Irritant	N	Drug likeness	−3.54
Reproductive effect	N	Drug score	0.45
-	-	nHBA & nHBD	1.0
-	-	cLogP	3.49
-	-	cLogS	−1.89

N = No risk, TPSA = topological polar surface area, cLogP = the partition coefficient between n-octanol/water, cLogS = the coefficient of solubility in water.

**Table 4:** In silico pkCSM pharmacokinetic parameters of geraniol.

Property	Model Name	Predicted Value	Unit
Absorption	Water solubility	−2.866	Numeric (log mol/L)
	Caco-2 permeability	1.49	Numeric (log Papp in 10^−6^ cm/s)
	Intestinal absorption (human)	92.788	Numeric (% Absorbed)
	Skin permeability	−1.511	Numeric (log Kp)
	P-glycoprotein substrate	No	Categorical (Yes/No)
	P-glycoprotein I inhibitor	No	Categorical (Yes/No)
	P-glycoprotein II inhibitor	No	Categorical (Yes/No)
Distribution	VDss (human)	0.17	Numeric (log L/kg)
	Fraction unbound (human)	0.447	Numeric (Fu)
	BBB permeability	0.606	Numeric (log BB)
	CNS permeability	−2.159	Numeric (log PS)
Metabolism	CYP2D6 substrate	No	Categorical (Yes/No)
	CYP3A4 substrate	No	Categorical (Yes/No)
	CYP1A2 inhibitior	No	Categorical (Yes/No)
	CYP2C19 inhibitior	No	Categorical (Yes/No)
	CYP2C9 inhibitior	No	Categorical (Yes/No)
	CYP2D6 inhibitior	No	Categorical (Yes/No)
	CYP3A4 inhibitior	No	Categorical (Yes/No)
Excretion	Total clearance	0.437	Numeric (log ml/min/kg)
	Renal OCT2 substrate	No	Categorical (Yes/No)
Toxicity	AMES toxicity	No	Categorical (Yes/No)
	Hepatotoxicity	No	Categorical (Yes/No)
	Oral rat acute toxicity	1.636	Numeric (mol/kg)

## Data Availability

Data are contained within the article and Supplementary Materials, and will be available from the corresponding author upon request.

## Supplementary Materials

The following supporting information can be downloaded at: https://www.mdpi.com/article/10.3390/pharmaceutics16081053/s1, Table S1: Characteristics of oligonucleotide primers used in this study; Table S2: Antifungal interaction between geraniol and itraconazole on planktonic cells of *Candida tropicalis*; Table S3: Binding energy and specific interaction of *Ct*Cdr1 with geraniol, curcumin and farnesol; Figure S1: Pattern of *CDR1* (black bars) and *ERG3* (white bars) expression in azole-resistant *Candida tropicalis*; Figure S2: Cell viability and plasma membrane integrity analyses of *Candida tropicalis* ATCC 28707 after differential labeling with FUN-1^™^ and Calcofluor White^™^ MR2; Figure S3: Kinetics of biofilm formation by azole-resistant *Candida tropicalis* on polystyrene surface monitored by measuring the metabolic activity of sessile cells using the MTT reduction assay (OD_550 nm_); Figure S4: Fungal load in hemolymph of *Galleria mellonella* larvae.
