# Supplementary material for: Geraniol Potentiates the Effect of Fluconazole against Planktonic and Sessile Cells of Azole-Resistant Candida tropicalis: In Vitro and In Vivo Analyses
Source: Pharmaceutics. 2024 Aug 9;16(8):1053. doi: 10.3390/pharmaceutics16081053 (PMC11360560; doi:10.3390/pharmaceutics16081053)
Supplement: Supplementary file 1 [file pharmaceutics-16-01053-s001.zip › pharmaceutics-3103335-supplementary.pdf]

Table S1: Characteristics of oligonucleotide primers used in this study

**Gene**	**Amplicon (pb)**	**Annealing temperature(ºC)**	***Primers* (5'-3') F/R**	**Reference**
CDR 1	169	57	TACATCAACCTTCTGCGCTTAT<br>TCTGCTGGATTGGCTTCTTT	This study
ERG 3	175	57	ACTGGTGGTTATAAGGCACTTT<br>CTGGATGGAAAGCATGGGAT	This study
MDR 1	315	55	CCCAGAAGTTTTCATTCCA<br>CCCCAAGCAACAGGATAAT	Bizerra *et al.* [10]
ERG 11	320	55	ATGGCTATTGTTGATACTGC<br>GCATTGTAAATGAATTCGTG	Bizerra *et al.* [10]
ACT 1	322	55	ATGGACGGGGGTATGTTTCA<br>GACATAAGTAATTTCCAATGTG	Bizerra *et al.* [10]

Table S2: Antifungal interaction between geraniol and itraconazole on planktonic cells of *Candida tropicalis*

Candida tropicalis	**GER MIC (µg/mL)**	**ITR MIC (µg/mL)**	**GER/ ITR MIC (µg/mL)**	**FICI**	**Interaction***
ATCC 28707	512	1	128/0.015	0.27	Synergism
CTR 1	512	2	128/0.015	0.26	Synergism
CTR 2	512	1	128/0.015	0.27	Synergism
CTR 3	512	1	128/0.015	0.27	Synergism

GER: Geraniol; ITR: Itraconazole; MIC: Minimum inhibitory concentration; FICI: Fractional inhibitory concentration index; *Reference values: synergism, FICI $\leq$ 0.5; antagonism, FICI $\geq$ 4 and indifferent, FICI >0.5 to 4 [42].

Table S3. Binding energy and specific interaction of *Ct*Cdr1 with geraniol, curcumin and farnesol

Compound	Δ*G* (kcal/mol)	Protein ligands interaction	
		**Hydrophobic**	**H-bonds**
Geraniol	-5.61	Gln131, Thr99, Asn97, Arg100, Gly789, Ser788, Ile816, Gly101, Leu102, Leu790, Tyr130, Glu785	Thr787, His817
	-5.51	Gln131, Leu790, Ser788, Gly789, Thr99, Tyr130	His817, Thr787
	-5.45	Gln131, His817, Tyr130, Gly787, Ser788, Asn97, Thr99, Leu790	Thr787
	-5.18	Thr99, Gln131, Gly789, Ser788, Thr787	His817
Curcumin	-9.55	Arg100, Ser788, Gly789, Gly101, Leu102, Ser104, Gln818, Ser133, Ser136, Thr99, Ile129, Cys132	Asn97, Gln131, His817
	-8.85	Ser788, Thr99, Ser104, Gln818, Ser820, Ser788, Gln131, Pro819	His817, Ser820, Thr787
	-7.85	Leu102, His817, Gln818, Ser133, Cys132, Ser820, Gln131, Tyr130, Thr787, Ile816, Ser788, Gly789, Thr176	Gln131, Thr99
	-7.30	Gln131, Ser104, Leu102, Asp103, Ala107, His817, Gly789, Ser788, Glu785, Gln700, Gly101	Ser133, Gln700, Gly789
Farnesol	-7.00	Gln818, Asp103, His817, Leu102, Ser788, Gly101, Tyr130, Arg100, Thr99, Gly787, Leu790, Asn97, Ile129, Ser104, Ser136, Cys132	Ser133, Gln131
	-6.90	His817, Ser104, Ser133, Gln131, Ser136, Ala107, Gly789, Tyr130	Asn97
	-6.21	Ser136, Ser133, Ser104, Leu102, Thr99, Gln131, Gly101, Asp103, His817, Gly789	Asn97
	-5.94	Ser104, Ala107, Leu102, Thr99, Gly101, Gln131, Asn97, Gly789, Arg100, Ser788, His817, Ser104	Thr99

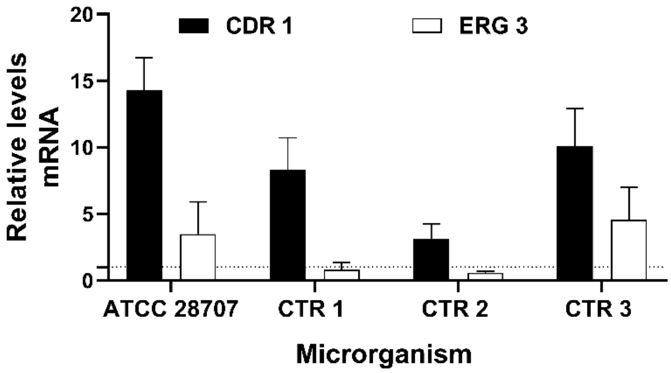

Figure S1: Pattern of *CDR1* (black bars) and *ERG3* (white bars) expression in azole-resistant *Candida tropicalis*. mRNAs from planktonic cells were obtained and the expression were quantified by real time PCR using SYBR green system and cycle threshold method. The height of the bars represents the fold change in gene expression planktonic cells incubated with 256 µg/mL fluconazole for 12 h compared to untreated ones. Bars represent data from three replicates. Error bars represent standard deviations.

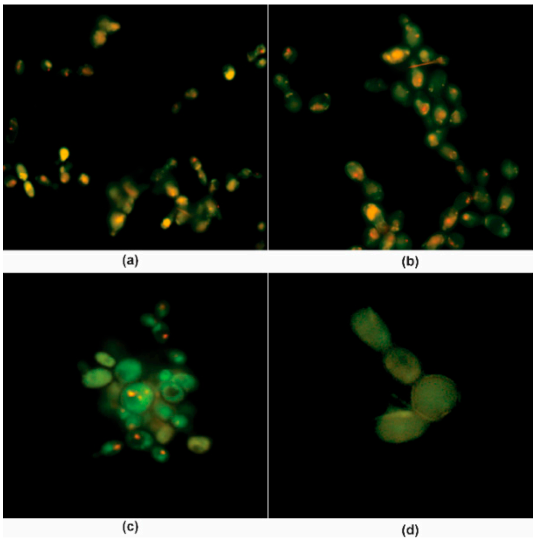

Figure S2: Cell viability and plasma membrane integrity analyses of *Candida tropicalis* ATCC 28707 after differential labeling with FUN-1™ and Calcofluor White™ MR2. Yeasts were incubated without (**a,b**) and with MIC of geraniol (**c,d**) for 4 h. **(a,b)** Cells containing red fluorescent intravacuolar structures represent metabolically active yeast and plasma membrane integrity. **(c,d)** Cells with diffuse greenish-yellow fluorescence characterize metabolically inactive cells.

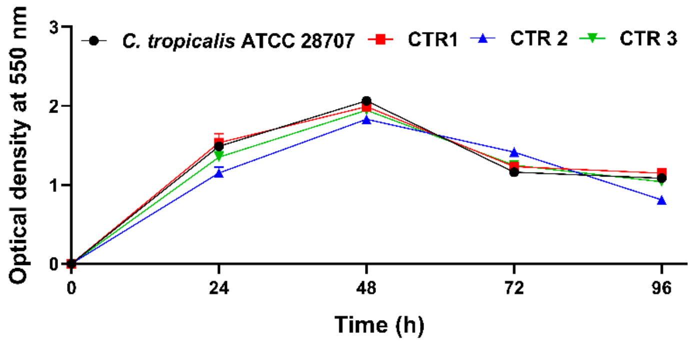

Figure S3: Kinetics of biofilm formation by azole-resistant *Candida tropicais* on polystyrene surface monitored by measuring the metabolic activity of sessile cells using the MTT reduction assay (OD_{550nm}). The values represent the mean ± standard deviation of three independent experiments.

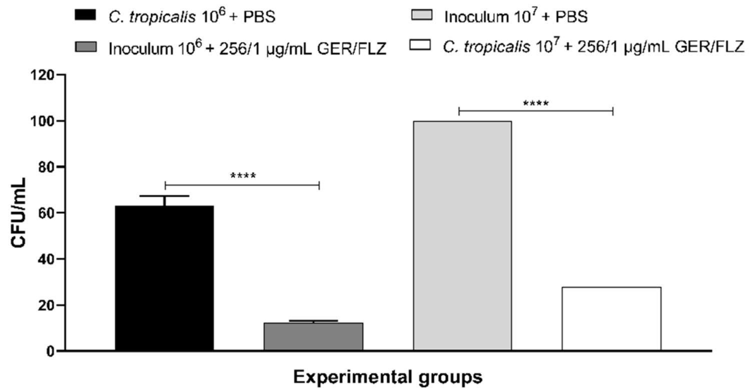

Figure S4: Fungal load in hemolymph of *Galleria mellonella* larvae. The larvae were infected with fungal cells (1×10^6 or 1×10^7) and were treated with GER/FLZ combination (256/1 µg/mL) after 2 h-post-infection. Fungal load was determined by colony forming unit (CFU) counts. **** $p<0.0001$ compared to GER/FLZ untreated groups.
